# Age-related behavioural abnormalities in C57BL/6.KOR–*Apoe* ^shl^ mice

**DOI:** 10.1515/tnsci-2022-0363

**Published:** 2025-02-25

**Authors:** Hiroshi Ueno, Yu Takahashi, Sachiko Mori, Eriko Kitano, Shinji Murakami, Kenta Wani, Tetsuji Miyazaki, Yosuke Matsumoto, Motoi Okamoto, Takeshi Ishihara

**Affiliations:** Department of Medical Technology, Kawasaki University of Medical Welfare, Okayama, 701-0193, Japan; Department of Psychiatry, Kawasaki Medical School, Kurashiki, 701-0192, Japan; Department of Neuropsychiatry, Graduate School of Medicine, Dentistry and Pharmaceutical Sciences, Okayama University, Okayama, 700-8558, Japan; Department of Medical Technology, Graduate School of Health Sciences, Okayama University, Okayama, 700-8558, Japan

**Keywords:** age, apolipoprotein, behavioural test, central nervous system, mouse

## Abstract

Spontaneously hyperlipidaemic (Apoe^shl^) mice were discovered in 1999 as mice lacking apolipoprotein E (ApoE) owing to a mutation in the *Apoe* gene. However, age-related behavioural changes in commercially available Apoe^shl^ mice have not yet been clarified. The behavioural abnormalities of ApoE-deficient mice, which are genetically modified mice artificially deficient in ApoE, have been investigated in detail, and it has been reported that they can serve as a model of Alzheimer’s disease (AD). To understand whether Apoe^shl^ mice can also serve as a murine model of AD, it is necessary to investigate age-related behavioural abnormalities in Apoe^shl^ mice. In this study, we conducted a series of behavioural experiments on 7- and 11-month-old Apoe^shl^ mice to investigate the behavioural abnormalities associated with ageing in Apoe^shl^ mice. In this study, 7-month-old Apoe^shl^ mice showed decreased body weight and grip strength compared to age-matched wild-type mice. In the open field test, 7-month-old Apoe^shl^ mice showed increased anxiety-like behaviour compared to wild-type mice, whereas 11-month-old Apoe^shl^ mice showed decreased anxiety-like behaviour. Moreover, Apoe^shl^ mice aged 7 and 11 months had increased serum cholesterol levels. These results indicate that the behaviour of Apoe^shl^ mice changes with age. However, 11-month-old Apoe^shl^ mice did not show a decline in cognitive function or memory ability similar to murine models of AD. Our findings indicate that Apoe^shl^ mice can be used to investigate the function of ApoE in the central nervous system.

## Abbreviations

AD: Alzheimer’s disease
ANOVA: analysis of variance
Apo: apolipoprotein
CNS: central nervous system
KO: knockout
SHL: spontaneously hyperlipidaemic

## 1 Introduction

Apolipoprotein E (ApoE) is a major apolipoprotein involved in the lipid metabolism of the central nervous system (CNS) [1]. In humans, three major polymorphic allelic variants of the *APOE* gene have been identified, and the resulting ApoE isoforms (ApoE2, ApoE3, and ApoE4) differ by a single amino acid. Consistent with the important role of ApoE in brain homeostasis and injury repair, *APOE* polymorphisms have been reported to be associated with Alzheimer’s disease (AD) and age-related cognitive decline [2–4]. ApoE isoforms have specific functions in regulating amyloid β aggregation and clearance in the brain and modulating several other pathologies implicated in AD, including diabetes mellitus [5].

The functions of ApoE and its isoforms extend beyond lipid metabolism and include maintaining normal brain function [6] as ApoE is expressed in both central and peripheral nervous systems [7]. ApoE is a major carrier of cholesterol required for neuronal activity and damage repair in the brain; therefore, it has been implicated in removing debris from damaged cells and stimulating neuronal regeneration [8]. ApoE knockout (KO) mice are widely used as an AD model. ApoE-deficient mice exhibit fewer huddled contacts during sleep, reduced motor activity in novel environments, and learning and memory impairments [9]. In mice, ApoE deficiency causes learning deficits in behavioural tasks related to hippocampal function [10,11]. These behavioural abnormalities are consistent with cognitive impairment and memory loss, which are the earliest clinical symptoms of neurodegenerative diseases including AD. Therefore, ApoE-deficient mice are considered one of the mouse models of AD [12].

Matsushima et al. discovered spontaneously hyperlipidaemic (SHL) mice during the process of generating inbred strains from wild-type mice [13]. Spontaneous hyperlipidaemia in SHL mice, a natural mutant, is caused by ApoE deficiency due to mutations in the *Apoe* gene [14]. The *Apoe* ^*shl*^ gene, which is responsible for hyperlipidaemia in this strain, was introduced into the genetic background of the atherosclerosis-susceptible C57BL/6 strain to generate B6.KOR/StmSlc-*Apoe* ^*shl*^ (B6.SHL) mice [14] which are commercially available. Although ApoE KO mice are the leading mouse model of AD, they are expensive to purchase from Jackson Laboratory in the United States, and it requires time to obtain them. Furthermore, experiments using ApoE-KO mice require laborious procedures in accordance with the Act on the Regulation of Genetically Modified Organisms. In contrast, SHL mice purchased from Japan SLC are naturally occurring ApoE-mutant animals; therefore, these regulations do not apply. Considering the above points, Apoe^shl^ mice represent a more natural model that probably better reflects pathological changes over time [14]. Thus, Apoe^shl^ mice, which spontaneously develop symptoms, are expected to be more useful models for hyperlipidaemia, atherosclerosis, and AD than ApoE-deficient mice. In a previous study, we reported that 10-week-old Apoe^shl^ mice show decreased motor learning and increased anxiety-like behaviour toward height [15]. However, age-related changes in behavioural and CNS abnormalities in Apoe^shl^ mice have not yet been investigated.

Ageing is associated with various behavioural changes that are mediated by alterations in brain networks [16,17]. It is a complex process that causes considerable structural and functional changes in the brain, leading to an increased incidence of neuropsychiatric and neurodegenerative disorders [18]. Numerous studies have investigated the effects of ageing on brain physiology and behaviour [19,20] and have demonstrated age-related impairments in cognitive function [21]. However, it remains unclear whether Apoe^shl^ mice develop age-related behavioural abnormalities.

The present study in Apoe^shl^ mice was conducted to investigate the effects of age on their behaviour from young adulthood to midlife and to identify age-related behavioural changes in their early life. We used a behavioural test battery to obtain behavioural data from Apoe^shl^ mice at 7 and 11 months of age. This study helps to understand the effects of ApoE on age-related behaviour and the CNS. Furthermore, this study will help identify new applications of the Apoe^shl^ mouse model.

## 2 Methods

### 2.1 Animals

Two-month-old C57BL/6.KOR–*ApoE* ^*shl*^ and wild-type C57BL/6N mice were purchased from Japan SLC (Shizuoka, Japan). Each strain was randomly divided into two groups, a 7- and an 11-month-old group (*n* = 10). These mice were kept in cages (five animals per cage) with food and water *ad libitum* under a 12 h light/dark cycle at 23–26°C until they were 7 or 11 months old. Since murine behaviour is partially sex-dependent and this study did not seek to compare sex differences, only male mice were included.

### 2.2 Behavioural tests

All behavioural tests were conducted between 09:00 and 16:00 in behavioural testing rooms. After each test, the equipment was cleaned with 70% ethanol and super-hypochlorous water to prevent artefacts caused by lingering olfactory cues. Behavioural tests were performed on naïve mice in the order described below. Each animal was used in more than one behavioural test.

### 2.3 Wire hang test

For the wire hang test, a wire hang test device (O’Hara & Co., Tokyo, Japan) was used. Each mouse was placed on top of the wire mesh which was turned over and gently shaken to encourage the mouse to grab the wire. Subsequently, the time until falling was recorded.

### 2.4 Grip strength test

Neuromuscular strength was examined using the grip strength test. Forelimb muscle strength was measured using a grip dynamometer. Each mouse was lifted by its tail so that its front paws could grip the wire grid of the dynamometer. Subsequently, the mouse was slowly pulled back until it released the grid. The peak force exerted by the forelimbs was recorded in Newton (cN).

### 2.5 Hot plate test

The hot plate test was used to assess nociception. Mice were placed on a plate heated to 55.0 ± 0.3°C, and the latency to the first paw response was recorded. Valid responses included shaking or licking the paw. A latency period of 30 s was defined as complete analgesia and was used as the cut-off time to prevent tissue damage.

### 2.6 Cotton bud biting test

Aggressive behaviour was tested using the cotton swab bite test. The mouse was placed in the experimenter’s hand, and a sterile cotton swab was applied near its face. Biting of the swab was considered aggressive. Each mouse was tested ten times. The total number of biting attacks was recorded for analysis.

### 2.7 Rotarod test

Motor alignment and balance were assessed using the rotarod test. In this test using an accelerating rotarod (RTR-M5, Melquest, Toyama, Japan), a mouse is placed on top of a rotating drum (3.9 cm in diameter), and the time the mouse is able to maintain balance on the rod is measured. The rotarod speed was increased from 4 to 40 rpm over 5 min. The trial interval for this test was 20 min. All mice were tested without prior training.

### 2.8 Elevated plus-maze test

Anxiety-like behaviour was investigated using the elevated plus-maze test. The maze device consisted of two opposing open arms (8 × 25 cm) with transparent walls and two opposing closed arms of the same size made of white plastic, all at a height of 40 cm above the ground. Each mouse was placed in the central square of the maze facing a closed arm and was allowed to move freely between the four arms for 6 min. The mice were video recorded. We used video tracking software (ANY-MAZE, Stoelting Co., Wood Dale, IL, USA) to analyse the number of arm entries, distance travelled (m), and time spent in the open arms.

### 2.9 Light/dark transition test

A light/dark transition test was used to examine anxiety-like behaviour. The device consisted of an acrylic cage (22 × 44 × 40 cm) divided into two equally sized sections by a partition with a door. One chamber had a white acrylic wall and was brightly lit (200 lx) with light placed above the ceiling of the chamber. The other chamber had black acrylic walls and was dark (50 lx). Both chambers contained white plastic floors. The mice were placed in the dark chamber and allowed to move freely between the two chambers for 6 min with the door open. ANY-MAZE software was used to analyse the distance travelled (m), total number of transitions, and time spent in the lit chamber(s).

### 2.10 Open field test

Open field tests were used to examine exploratory, anxiety-like, and general motor activity. Each mouse was placed in the centre of the apparatus, which consisted of a square area (45 × 45 × 40 cm) surrounded by walls. The distance travelled (m), number of entries into the central area, and time spent in the central area (s) were recorded. The central region was defined as the central 20 × 20 cm section. The test chamber was illuminated at 100 lx. Data were collected over 30 min. Data analysis was performed using the ANY-MAZE software.

### 2.11 Y-maze test

Spatial working memory was measured using a Y-maze apparatus (arm length, 40 cm; bottom arm width, 3 cm; upper arm width, 10 cm; wall height, 12 cm). Each mouse was placed in the centre of the Y-maze for 6 min. Visual cues were placed around the maze in the testing chamber and remained there throughout the test period. The mice were tested without any previous exposure or habituation to the maze. The total distance travelled (m), number of entries, and number of turnovers were recorded and analysed using ANY-MAZE software.

### 2.12 Passive avoidance test

A two-compartment, step-through, passive avoidance device (MPB-M020; Melquest) was used. The apparatus was divided into a light compartment (9.0 × 18.0 × 14.5 cm) and a dark compartment (18.0 × 18.0 × 14.5 cm) by a wall with a guillotine door. The bright compartment was illuminated with fluorescent light (200 lx). The mice were placed in the bright compartment and allowed to explore it for 20 s. Subsequently, the guillotine door was raised to allow the mouse to enter the dark room and closed once inside. Subsequently, an electric shock (0.5 mA) was applied to their feet for 3 s. The test session was conducted 24 h after the training session. The latency to enter the dark room was recorded for up to 180 s.

### 2.13 Tail suspension test

Depression-like behaviour was assessed using the tail suspension test. Each mouse was suspended by its tail 60 cm above the floor in a white plastic chamber using adhesive tape placed less than 1 cm from the tail end. The resulting behaviour was recorded for 6 min. A video camera was used to capture images and measure the immobility time. The “immobility period” was defined as the interval in which the animal stopped struggling for more than 1 s. Data acquisition and analysis were performed using the ANY-MAZE software.

### 2.14 Porsolt forced swim test

Depression-like behaviour was assessed using the Porsolt forced swim test. The device consisted of four Plexiglass cylinders (20 cm height × 10 cm diameter). The cylinder was filled with water (23°C) to a depth of 7.5 cm. The mice were placed inside the cylinder for 6 min, and their behaviour was recorded. Similar to the tail suspension test, the immobility time was assessed using ANY-MAZE software.

### 2.15 Serum cholesterol analysis

At the end of the behavioural experiments, blood samples were collected by puncturing the left ventricle immediately before euthanasia. The blood samples were allowed to clot. The serum was immediately separated by centrifugation at 1,700 × *g* for 10 min and stored at −80°C. Serum total cholesterol levels were measured using a LabAssay^TM^ Cholesterol (LABCHO-M1; FUJIFILM Wako Shibayagi, Gunma, Japan).

### 2.16 Statistical analyses

Statistical analyses were conducted using the SPSS software (IBM Corp., Tokyo, Japan). Normal distribution was determined using the Shapiro–Wilk normality test for all samples before any group analysis. For normally distributed paired samples, a paired *t*-test was used. For non-normally distributed paired samples, we used the Mann–Whitney *U* test. One-way analysis of variance (ANOVA) followed by Tukey’s test was used to compare two experimental groups in which unpaired samples were normally distributed. We used the Kruskal–Wallis test to compare two experimental groups, in which unpaired samples were not normally distributed. The data are presented as box plots. Statistical significance was defined as *p < 0.05 and ^+^ *p* < 0.05.


**Ethical approval:** The research related to animals’ use has been complied with all the relevant national regulations and institutional policies for the care and use of animals. All animal experiments were performed following the ARRIVE guidelines (https://www.nc3rs.org.uk/arrive-guidelines) and the U.S. National Institutes of Health (NIH) Guide for the Care and Use of Laboratory Animals (NIH Publication No. 80-23, revised in 1996) and were approved by the Committee for Animal Experiments at Kawasaki Medical School Advanced Research Centre. All efforts were made to minimise the number of animals used and their suffering. The required sample size was calculated using power analysis.

## 3 Results

### 3.1 Body weight and grip strength are decreased in 7-month-old Apoe^shl^ mice

**Figure 1:**
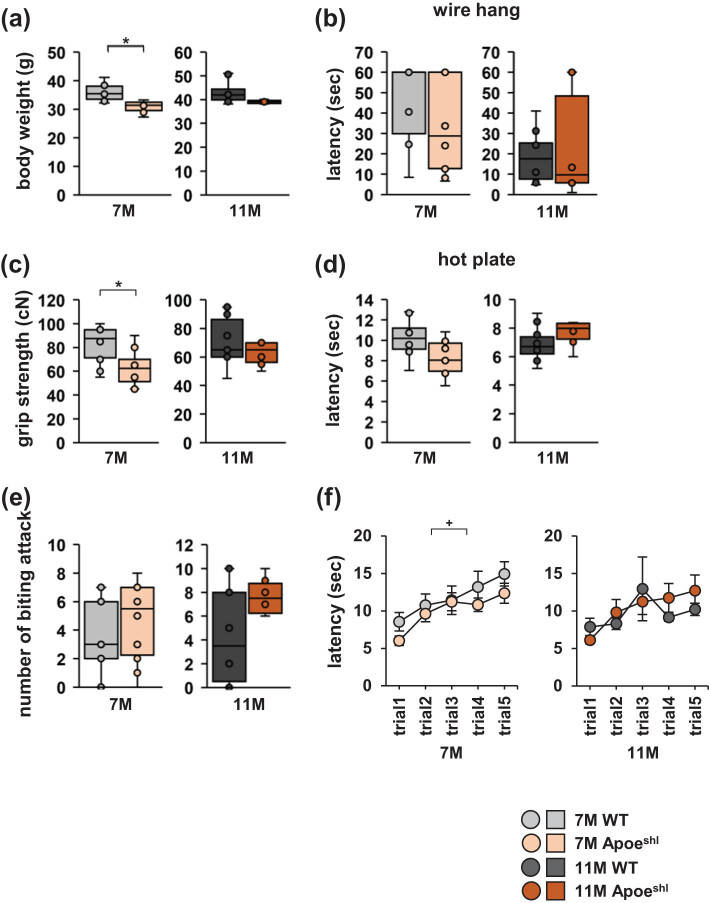
Physical characteristics of 7- and 11-month-old Apoe^shl^ mice. (a) Body weight. (b) Latency to fall in the wire hang test. (c) Grip strength. (d) Hot plate test. (e) Number of times biting on the cotton bud. (f) Latency to fall in the rotarod test. Data are presented as box plots (a–e) or mean ± standard error (f). Statistical significance is indicated by asterisks: *p < 0.05, ^+^ *p* < 0.1. The *p*-values were calculated using one-way ANOVA (a–e) or two-way repeated-measures ANOVA (f). (a–f) 7-month-old wild-type (WT): *n* = 10, 11-month-old wild-type (WT): *n* = 10, 7-month-old Apoe^shl^: *n* = 10, 11-month-old Apoe^shl^: *n* = 10.

Both 7- and 11-month-old Apoe^shl^ mice appeared healthy, with no obvious differences in physical characteristics compared with wild-type mice. The body weight of 7-month-old Apoe^shl^ mice was significantly lower than that of wild-type mice (Figure 1a, one-way ANOVA: 7-month-old, *F*_1,18_ = 19.707, *p* < 0.001*). The body weight of 11-month-old Apoe^shl^ mice was not significantly different from that of wild-type mice (Figure 1a, one-way ANOVA: 7-month-old, *F*_1,18_ = 1.857, *p* = 0.194). The time in the wire hang test was not significantly different between Apoe^shl^ and wild-type mice at both 7 and 11 months of age (Figure 1b, one-way ANOVA: 7-month-old, *F*_1,18_ = 1.555, *p* = 0.228; 11-month-old, *F*_1,18_ = 0.349, *p* = 0.564). Grip strength was in 7-month-old Apoe^shl^ mice significantly lower than in age-matched wild-type mice (Figure 1c, one-way ANOVA: 7-month-old, *F*_1,18_ = 7.521, *p* = 0.013; 11-month-old, *F*_1,18_ = 0.152, *p* = 0.702). At 7 and 11 months of age, the pain threshold measured using the hot plate test was not significantly different between the Apoe^shl^ and wild-type groups (Figure 1d, one-way ANOVA: 7-month-old, *F*_1,18_ = 1.194, *p* = 0.289; 11-month-old, *F*_1,18_ = 2.544, *p* = 0.133). Likewise, there was no significant group difference in the number of bite attacks in the cotton bud biting test (Figure 1e, one-way ANOVA: 7-month-old, *F*_1,18_ = 0.639, *p* = 0.434; 11-month-old, *F*_1,18_ = 1.653, *p* = 0.219). As shown in Figure 1f, 7-month-old Apoe^shl^ mice showed a trend toward a decreased latency to fall in the rotarod test (Figure 1f, two-way repeated-measures ANOVA: 7-month-old, group × time: *F*_4,90_ = 3.921, *p* = 0.051^+^). At 11 months of age, the latency to fall in the rotarod test was not significantly different between the groups (Figure 1f, two-way repeated-measures ANOVA: 11-month-old, group × time: *F*_4,90_ = 0.222, *p* = 0.639).

### 3.2 In the elevated plus-maze test, 7- and 11-month-old Apoe^shl^ mice do not show anxiety-like behaviour

**Figure 2:**
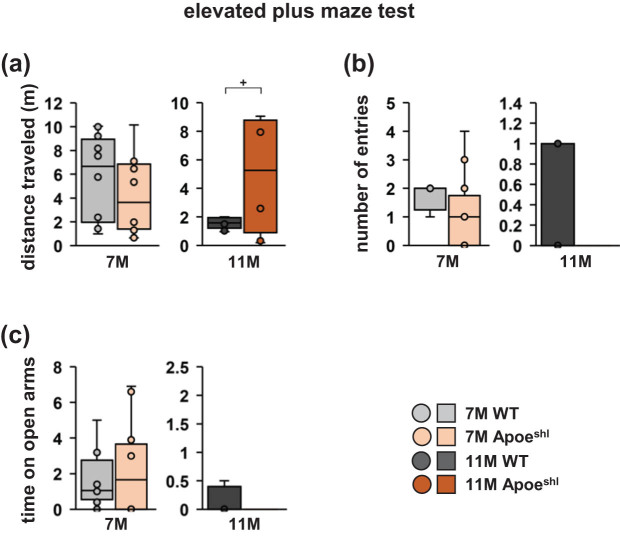
Elevated plus-maze test performance of 7- and 11-month-old Apoe^shl^ mice. Elevated plus-maze test: total distance travelled (a), number of total entries into open arms (b), and time spent in the open arms (c). Data are presented as box plots (a–c). Statistical significance is indicated by asterisks: *p < 0.05, ^+^ *p* < 0.1. The *p*-values were calculated using one-way ANOVA (a–c). (a–c) 7-month-old wild-type (WT): *n* = 10, 11-month-old wild-type (WT): *n* = 10, 7-month-old Apoe^shl^: *n* = 10, 11-month-old Apoe^shl^: *n* = 10.

Anxiety-like behaviour was assessed using the elevated plus-maze test. There were no differences between groups in total distance travelled (Figure 2a, one-way ANOVA: 7-month-old, *F*_1,18_ = 0.891, *p* = 0.358; 11-month-old, *F*_1,18_ = 3.363, *p* = 0.088^+^), total number of open arm entries (Figure 2b, one-way ANOVA: 7-month-old, *F*_1,18_ = 1.455, *p* = 0.243; 11-month-old, *F*_1,18_ = 0.714, *p* = 0.412), and time spent in open arms (Figure 2c, one-way ANOVA: 7-month-old, *F*_1,18_ = 0.070, *p* = 0.794; 11-month-old, *F*_1,18_ = 0.556, *p* = 0.468).

### 3.3 In the light/dark transition test, 7-month-old Apoe^shl^ mice show a reduced distance travelled

**Figure 3:**
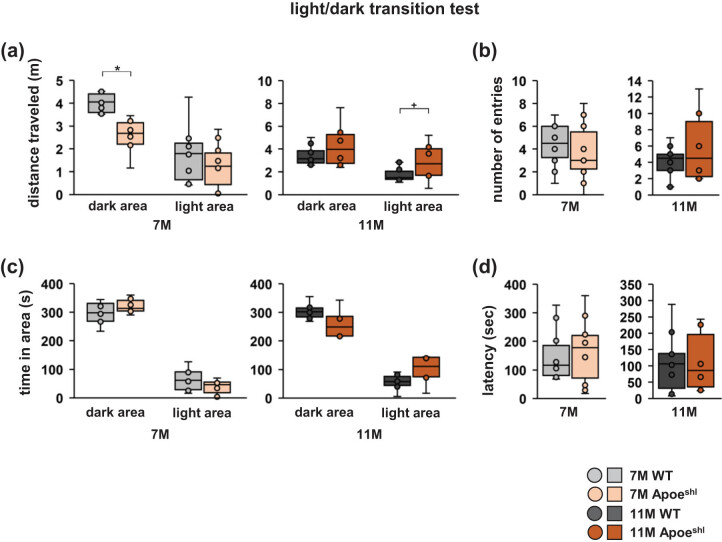
Light/dark transition test performance of 7- and 11-month-old Apoe^shl^ mice. Light/dark transition test: distance travelled in the dark and light areas (a), number of light/dark transitions (b), time spent in the dark and light areas (c), and latency to first transition to the light area (d). Data are presented as box plots (a–d). Statistical significance is indicated by asterisks: *p < 0.05, ^+^ *p* < 0.1. The *p*-values were calculated using two-way ANOVA (a, c) or one-way ANOVA (b, d). (a–d) 7-month-old wild-type (WT), *n* = 10; 11-month-old wild-type (WT), *n* = 10; 7-month-old Apoe^shl^, *n* = 10; 11-month-old Apoe^shl^, *n* = 10.

In the light/dark transition test, 7-month-old Apo^shl^ mice travelled a decreased distance in the dark area (Figure 3a, two-way ANOVA: 7-month-old, group × area: *F*_1,36_ = 4.984, *p* = 0.032*; dark area, *p* = 0.026*; light area, *p* = 0.410). In contrast, the distance travelled in the light and dark areas was not significantly different between the 11-month-old groups (Figure 3a, two-way ANOVA: 11-month-old, group × area: *F*_1,36_ = 4.880, *p* = 0.036*; dark area, *p* = 0.189; light area, *p* = 0.086^+^). Moreover, no significant differences were observed between groups at 7 months of age regarding the number of transitions between light and dark compartments (Figure 3b, one-way ANOVA: 7-month-old, *F*_1,18_ = 1.174, *p* = 0.293), time spent in the light and dark areas (Figure 3c, two-way ANOVA: 7-month-old, group × area: *F*_1,36_ = 0.000, *p* = 1.0; dark area, *p* = 0.477; light area, *p* = 0.477), and latency to first transition to the light area (Figure 3d, one-way ANOVA: 7-month-old, *F*_1,18_ = 0.172, *p* = 0.683). Likewise, no significant differences were found between groups at 11 months of age (Figure 3b, one-way ANOVA: 11-month-old, *F*_1,18_ = 1.479, *p* = 0.244; Figure 3c, two-way ANOVA: 11-month-old, group × area: *F*_1,36_ = 0.000, *p* = 1.0; dark area, *p* = 0.189; light area, *p* = 0.189; Figure 3d, one-way ANOVA: 11-month-old, *F*_1,18_ = 0.016, *p* = 0.902).

### 3.4 In the open field test, 7-month-old Apoe^shl^ mice show a reduced distance travelled, whereas 11-month-old Apoe^shl^ mice show an increased distance travelled

**Figure 4:**
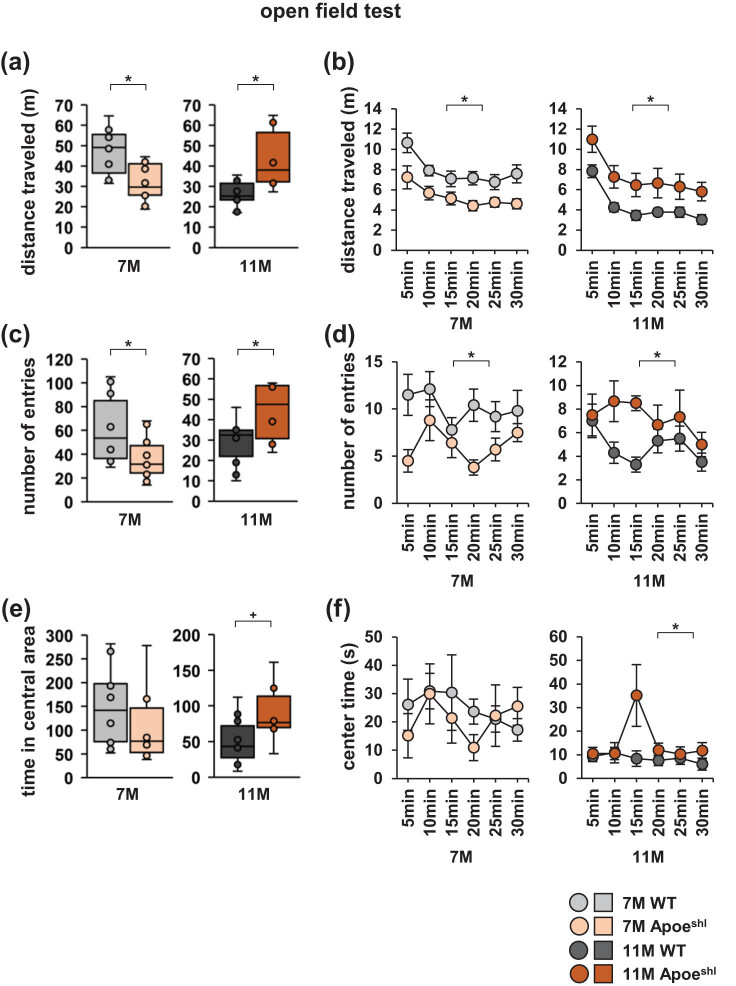
Open field test performance of 7- and 11-month-old Apoe^shl^ mice. Open field test: total distance travelled (a), number of total entries into the central area (c), total time spent in the central area (e), distance travelled every 5 min (b), number of entries into the central area every 5 min (d), and time spent in the central area every 5 min (f). Data are presented as box plots (a, c, e) or mean ± standard error (b, d, f). Statistical significance is indicated by asterisks: *p < 0.05, ^+^ *p* < 0.1. The *p*-values were calculated using one-way ANOVA (a, c, e) or two-way repeated-measures ANOVA (b, d, f). (a–f) 7-month-old wild-type (WT), *n* = 10; 11-month-old wild-type (WT), *n* = 10; 7-month-old Apoe^shl^, *n* = 10; 11-month-old Apoe^shl^, *n* = 10.

In the open field test, the total distance travelled by 7-month-old Apoe^shl^ mice was significantly lower than that travelled by wild-type mice (Figure 4a, one-way ANOVA: 7-month-old, *F*_1,18_ = 10.455, *p* = 0.005*). The distance travelled in each 5 min period was also lower in 7-month-old Apoe^shl^ mice than in wild-type mice (Figure 4b, two-way repeated-measures ANOVA: 7-month-old, group × time: *F*_4,90_ = 36.464, *p* < 0.001*). In contrast, the total distance travelled by 11-month-old Apoe^shl^ mice was significantly greater than that travelled by wild-type mice (Figure 4a, one-way ANOVA: 11-month-old, *F*_1,18_ = 9.608, *p* = 0.008*), which was also observed in each 5 min period (Figure 4b, two-way repeated-measures ANOVA: 11-month-old, group × time: *F*_4,90_ = 39.725, *p* < 0.001*). Compared to age-matched wild-type mice, 7-month-old Apoe^shl^ mice had a significantly lower number of central area entries in total (Figure 4c, one-way ANOVA: 7-month-old, *F*_1,18_ = 4.815, *p* = 0.042*) and in each 5 min period (Figure 4d, two-way repeated-measures ANOVA: 7-month-old, group × time: *F*_4,90_ = 18.359, *p* < 0.001*). In contrast, the total number of entries into the central area in 11-month-old Apoe^shl^ mice was significantly higher than that in wild-type mice (Figure 4c, one-way ANOVA: 11-month-old, *F*_1,18_ = 4.896, *p* = 0.044*), and this was also observed in each 5 min period (Figure 4d, two-way repeated-measures ANOVA: 11-month-old, group × time: *F*_4,90_ = 11.467, *p* = 0.001*). The total time spent in the central area did not significantly differ between 7-month-old Apoe^shl^ mice and wild-type mice (Figure 4e, one-way ANOVA: 7-month-old, *F*_1,18_ = 0.293, *p* = 0.595). The time spent in the central area during each 5 min period was also not significantly different in this age group (Figure 4f, two-way repeated-measures ANOVA: 7-month-old, group × time: *F*_4,90_ = 0.735, *p* = 0.393). The total time spent in the central area by 11-month-old Apoe^shl^ mice was not significantly different from that of wild-type mice (Figure 4e, one-way ANOVA: 11-month-old, *F*_1,18_ = 4.011, *p* = 0.065^+^). However, the time spent in the central area in each 5 min period was higher in 11-month-old Apoe^shl^ mice than in wild-type mice (Figure 4f, two-way repeated-measures ANOVA: 11-month-old, group × time: *F*_4,90_ = 7.347, *p* = 0.008*).

### 3.5 The number of arm entries in the Y-maze test is decreased in 7-month-old Apoe^shl^ mice

**Figure 5:**
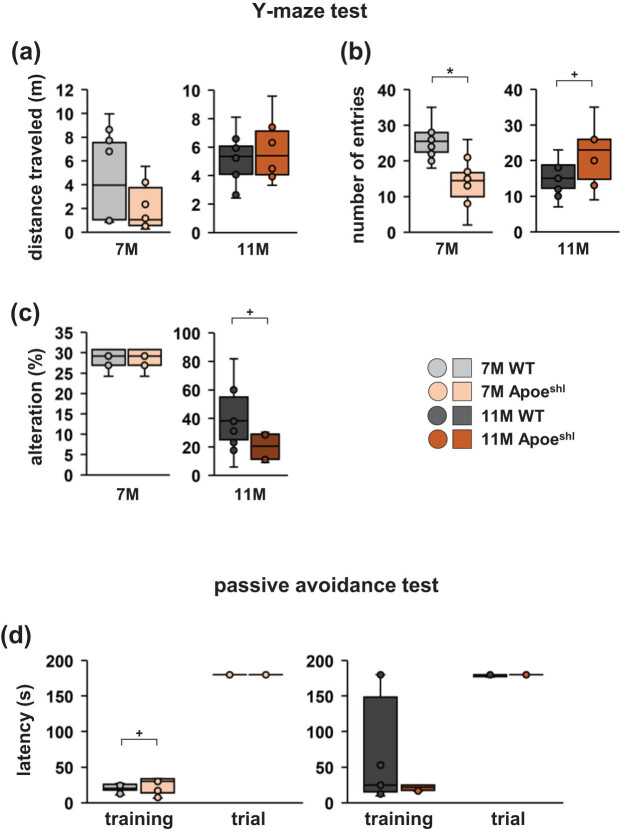
Y-maze test and passive avoidance test performance of 7- and 11-month-old Apoe^shl^ mice. Y-maze test: total distance travelled (a), total number of arm entries (b), and percentage of alternations (c). Passive avoidance test: the escape latencies during the training session and retention test (d). Data are presented as box plots (a–d). Statistical significance is indicated by asterisks: *p < 0.05, ^+^ *p* < 0.1. The *p*-values were calculated using one-way ANOVA (a–c) or two-way ANOVA (d). (a–d) 7-month-old wild-type (WT): *n* = 10, 11-month-old wild-type (WT): *n* = 10, 7-month-old Apoe^shl^: *n* = 10, 11-month-old Apoe^shl^: *n* = 10.

In the Y-maze test, no significant difference in the total distance travelled was found between Apoe^shl^ and wild-type groups at both 7 and 11 months of age (Figure 5a, one-way ANOVA: 7-month-old, *F*_1,18_ = 1.854, *p* = 0.190; 11-month-old, *F*_1,18_ = 0.548, *p* = 0.471). In 7-month-old Apoe^shl^ mice, the number of arm entries was significantly reduced compared to that in wild-type mice (Figure 5b, one-way ANOVA: 7-month-old, *F*_1,18_ = 18.438, *p* < 0.001*). This parameter was not significantly different between 11-month-old Apoe^shl^ and wild-type mice (Figure 5b, one-way ANOVA: 11-month-old, *F*_1,18_ = 3.256, *p* = 0.093^+^). Moreover, no significant difference in the percentage of alternations between groups was found at either age (Figure 5c, one-way ANOVA: 7-month-old, *F*_1,18_ = 0.1, *p* = 1.0; 11-month-old, *F*_1,18_ = 3.934, *p* = 0.067^+^).

### 3.6 Normal learning and memory in 7- and 11-month-old Apoe^shl^ mice in the passive avoidance test

In the step-through passive avoidance test, the 7-month-old groups showed no significant differences in latency to enter the dark room throughout the conditioning session (Figure 5d, two-way ANOVA: 7-month-old, group × time: *F*_1,36_ = 1.651, *p* = 0.207; training, *p* = 0.072^+^; trial, *p* = 0.970). After 24 h, the step-through latencies during the retention trials were also not significantly different between the 7-month-old groups (Figure 5d, two-way ANOVA: 7-month-old, group × time: *F*_1,36_ = 0.668, *p* = 0.421; training, *p* = 0.110; trial, *p* = 0.625). Likewise, no significant differences between the groups at 11 months of age were found in this test.

### 3.7 7- and 11-month-old Apoe^shl^ mice in the tail suspension test and Porsolt forced swim test

**Figure 6:**
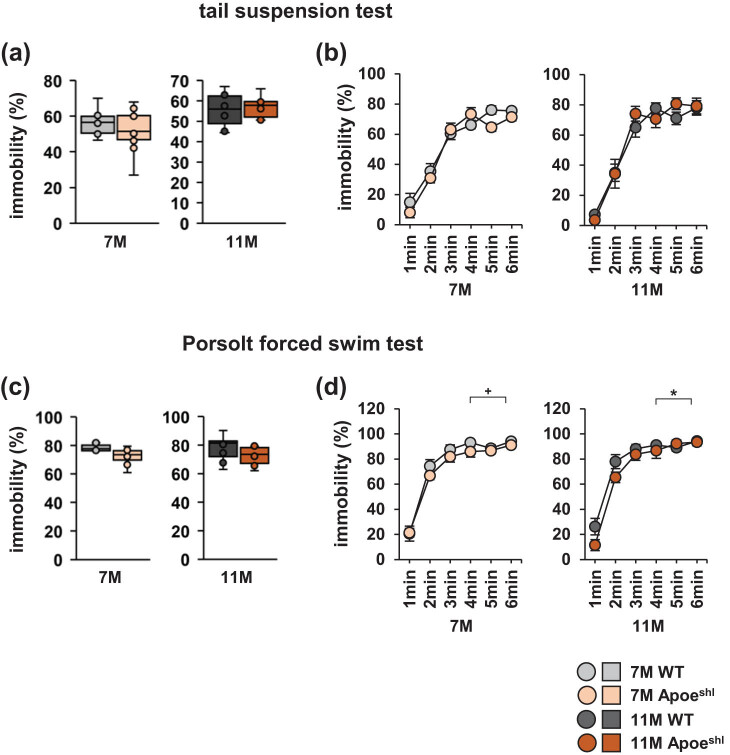
Tail suspension test and Porsolt forced swim test performance of 7- and 11-month-old Apoe^shl^ mice. Tail suspension test: the proportion of total time spent immobile (a) and the proportion of time spent immobile in each 1 min period (b). Porsolt forced swim test: the proportion of total time spent immobile (c) and the proportion of time spent immobile in each 1 min period (d). Data are presented as box plots (a, c) or mean ± standard error (b, d). Statistical significance is indicated by asterisks: *p < 0.05, ^+^ *p* < 0.1. The *p*-values were calculated using one-way ANOVA (a, c) or two-way repeated-measures ANOVA (b, d). (a–d) 7-month-old wild-type (WT): *n* = 10, 11-month-old wild-type (WT): *n* = 10, 7-month-old Apoe^shl^: *n* = 10, 11-month-old Apoe^shl^: *n* = 10.

In the tail suspension test, the total immobility time or percentage of time spent immobile per minute did not significantly differ between 7-month-old Apoe^shl^ mice and wild-type mice (Figure 6a, one-way ANOVA: 7-month-old, *F*_1,18_ = 0.354, *p* = 0.560; Figure 6b, two-way repeated-measures ANOVA: 7-month-old, group × time: *F*_5,108_ = 0.699, *p* = 0.405). Likewise, these parameters did not significantly differ between 11-month-old Apoe^shl^ mice and wild-type mice (Figure 6a, one-way ANOVA: 11-month-old, *F*_1,18_ = 0.146, *p* = 0.708; Figure 6b, two-way repeated-measures ANOVA: 11-month-old, group × time: *F*_5,108_ = 0.227, *p* = 0.635). In the Porsolt forced swim test, neither the total immobility time nor the percentage of immobility time for each 1 min differed significantly between 7-month-old Apoe^shl^ mice and wild-type mice (Figure 6c, one-way ANOVA: 7-month-old, *F*_1,18_ = 2.583, *p* = 0.125; Figure 6d, two-way repeated-measures ANOVA: 7-month-old, group × time: *F*_5,108_ = 3.633, *p* = 0.059^+^). Although the total immobility time per minute did also not significantly differ between 11-month-old Apoe^shl^ mice and wild-type mice (Figure 6c, one-way ANOVA: 11-month-old, *F*_1,18_ = 1.829, *p* = 0.198), the percentage of time spent immobile was significantly reduced in 11-month-old Apoe^shl^ mice compared to wild-type mice (Figure 6d, two-way repeated-measures ANOVA: 11-month-old, group × time: *F*_5,108_ = 4.832, *p* = 0.031*).

### 3.8 7- and 11-month-old Apoe^shl^ mice show an increased serum total cholesterol levels

**Figure 7:**
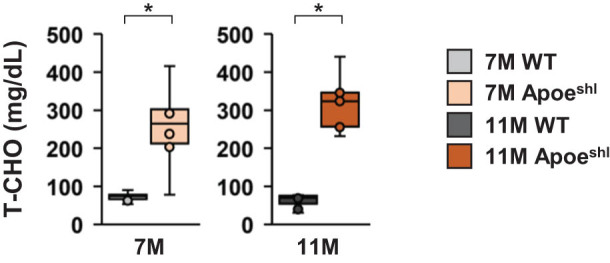
Serum total cholesterol levels of 7- and 11-month-old Apoe^shl^ mice. Comparison of serum total cholesterol levels. Data are presented as box plots. Statistical significance is indicated by asterisks: *p < 0.05, ^+^ *p* < 0.1. The *p*-values were calculated using one-way ANOVA. 7-month-old wild-type (WT): *n* = 10, 11-month-old wild-type (WT): *n* = 10, 7-month-old Apoe^shl^: *n* = 10, 11-month-old Apoe^shl^: *n* = 10.

We also investigated the serum total cholesterol levels in 7- and 11-month-old Apoe^shl^ mice (Figure 7). Compared to age-matched wild-type mice, 7-month-old Apoe^shl^ mice had significantly higher serum levels of total cholesterol (Figure 7, one-way ANOVA: 7-month-old, *F*_1,18_ = 18.454, *p* = 0.001*). Likewise, serum total cholesterol levels were significantly higher in 11-month-old Apoe^shl^ mice than in wild-type mice (Figure 7, one-way ANOVA: 11-month-old, *F*_1,18_ = 78.958, *p* < 0.001*).

## 4 Discussion

The findings of this study demonstrate behavioural abnormalities in Apoe^shl^ mice at 7 and 11 months of age. Compared to wild-type mice, 7-month-old Apoe^shl^ mice exhibited reduced body weight, reduced forelimb grip strength, and increased anxiety-like behaviour in the open field test. Moreover, 11-month-old Apoe^shl^ mice showed reduced anxiety-like behaviour in the open field test compared to wild-type mice. Apoe^shl^ mice at 7 and 11 months of age had also higher serum total cholesterol levels than wild-type mice. Interestingly, no persistent behavioural abnormalities were observed in Apoe^shl^ mice with aging.

In this study, 7-month-old Apoe^shl^ mice had significantly lower body weights than age-matched wild-type mice. However, there was no significant difference in body weight between 11-month-old Apoe^shl^ and wild-type mice. C57BL/6 mice gain weight with age [22]. Previous studies have reported that Apoe^shl^ mice have approximately the same body weight as wild-type mice until they are ∼2 months old [23] and that the body weight of Apoe^shl^ mice decreases subsequently compared to that of wild-type mice [24]. Deficiency or abnormality of ApoE causes hyperlipoproteinemia type III, which is characterised by early atherosclerosis and cholesterol accumulation in the blood. ApoE-deficient mice develop severe hypercholesterolaemia and atherosclerotic lesions resembling human lesions [25,26]. The decrease in body weight of 7-month-old Apoe^shl^ mice in the present study was thought to be due to abnormalities in lipid metabolism.

Compared to wild-type mice, 7-month-old Apoe^shl^ mice showed reduced forelimb grip strength. A previous study using 2-month-old Apoe^shl^ mice also showed a tendency for increased forelimb grip strength compared to wild-type mice, although no significant differences were found in the wire hang test [15]. However, this study showed no significant difference in grip strength between 11-month-old Apoe^shl^ and wild-type mice. Assessing muscle strength is an important aspect when studying neuromuscular disorders in rodent models [27]. Mice lose muscle strength after 3 months of age [22]. The ε4 allele of the *APOE* gene (*APOE4*) is associated with decreased muscle strength [28]. The present study shows that ApoE deficiency in mice causes muscle weakness from adulthood to middle age. Further research is required to investigate the mechanisms by which ApoE deficiency is associated with muscle strength.

In the rotarod test, 7-month-old Apoe^shl^ mice tended to show reduced locomotor performance compared to wild-type mice. Similarly, a previous study using 2-month-old Apoe^shl^ mice reported a significant reduction in their rotarod test scores compared to those of wild-type mice [15]. However, the performance of 11-month-old Apoe^shl^ mice in the rotarod tests of the present study was almost the same as that of wild-type mice. Motor function is normal in 12-month-old ApoE-deficient mice [9]. However, the ε4 allele has been reported to be associated with more rapid decline in exercise capacity in older adults [28]. The reduced exercise performance of Apoe^shl^ mice in the rotarod test may be due to differences in body weight. The results of the rotarod test are influenced by several factors, including motor coordination, learning, and cardiorespiratory endurance [29]. Although the mechanism underlying the association between reduced locomotor performance and ApoE is unknown, it has been speculated that ApoE deficiency may damage the locomotor system in mice at a relatively young age.

The ε4 allele has been reported to be associated with aggressive behaviour in older adults [30]. However, ApoE-deficient mice exhibit less aggressive behaviour than wild-type mice [31]. In the present study, 7- and 11-month-old Apoe^shl^ mice showed no changes in aggressive behaviour compared with wild-type mice. The results of this experiment show that Apoe^shl^ mice do not exhibit aggressive behaviour similar to ApoE-deficient mice.

Ageing is a known risk factor for degenerative changes in various regions of the brain [32]. Anxiety- and depression-like behaviours have been shown to increase significantly, whereas spatial learning and memory have been shown to be impaired [19,33,34]. In the early stages, people with AD may exhibit memory problems and depressive behaviour [35]. ApoE-deficient mice exhibit anxiety-like behaviour in the elevated plus-maze test compared to wild-type mice [36]. In this study, Apoe^shl^ mice at 7 and 11 months of age showed no behavioural abnormalities in the elevated plus-maze test compared with wild-type mice. However, 7-month-old Apoe^shl^ mice showed increased anxiety-like behaviour in light/dark traffic and open field tests compared to wild-type mice. In contrast, 11-month-old Apoe^shl^ mice showed reduced anxiety-like behaviour in the open field test compared to wild-type mice. Ageing is a complex process [37], and it has recently been argued that ageing can occur asynchronously and nonlinearly across cells, tissues, and time [38]. Anxiety is the most common symptom in AD patients with an onset age below 65 years [39]. In mice, several types of anxiety-like behaviour have been described, including anxiety evoked by heights, bright light, large objects, and wide open spaces [40]. Increased anxiety-like behaviour has previously been described in 2-month-old Apoe^shl^ mice compared to wild-type mice [15]. Our results showed that in Apoe^shl^ mice, anxiety evoked by large spaces changed significantly from 7 to 11 months of age. Anxiety-like behaviour increases with age in wild-type C57BL/6 mice [41]. In addition to lipid metabolism, ApoE and its role in maintaining normal brain function are also important [6]. Cholesterol dysfunction in the CNS may be associated with aging and the development of certain neurodegenerative diseases [42,43]. As observed in this study, the age-related changes in anxiety-like behaviour in Apoe^shl^ mice indicate that ApoE primarily influences emotional behaviour. Our results show that Apoe^shl^ mice exhibit age-related changes in anxiety-like behaviour which differ from those of wild-type mice.

In the Y-maze test, 7- and 11-month-old Apoe^shl^ mice showed no changes in cognitive function; however, the number of arm entries changed with age. Although 11-month-old Apoe^shl^ mice showed a tendency toward lower alternation rates in the Y-maze test, no significant difference was observed. It has been reported that 12-month-old ApoE-deficient mice exhibit decreased cognitive function in the Y-maze test [9]. On the other hand, ApoE-deficient mice showed better performance in spatial tasks in some studies [44,45]. These discrepancies may be related to mouse age, environmental factors, compensation by other proteins, genetic background, and the protocols and behavioural tasks employed [46,47]. It has also been reported that 12-month-old ApoE-deficient mice exhibit memory impairment in the passive avoidance test [9]. In the present study, 7- and 11-month-old Apoe^shl^ mice showed no behavioural abnormalities compared to wild-type mice in the passive avoidance test. Our results show that age-related behavioural changes differ between Apoe^shl^ and ApoE-deficient mice. Moreover, our findings demonstrate that the cognitive function of Apoe^shl^ mice changes with age. Further research is required to understand the details of these changes in cognitive function and memory in Apoe^shl^ mice over 11 months of age.

In humans, an association between ApoE gene polymorphism and susceptibility to depression has been reported [48]. Apoe^shl^ mice at 7 and 11 months of age did not exhibit increased depression-like behaviour in the tail suspension test. A previous study on 2-month-old Apoe^shl^ mice also did not report an increase in depression-like behaviour [15]. In the forced swim test, Apoe^shl^ mice at 11 months of age show decreased depression-like behaviour. There have been no prior reports of increased depression-like behaviour in the tail suspension or forced swimming tests in ApoE-deficient mice. The mechanism by which 11-month-old Apoe^shl^ mice showed a tendency toward reduced depressive-like behaviour in this study is unclear. This study shows that Apoe^shl^ mice exhibit different age-related behavioural changes from wild-type mice.

In this study, Apoe^shl^ mice at 7 and 11 months of age showed increased serum total cholesterol levels. No change in body weight was observed in Apoe^shl^ at 11 months of age, suggesting that the mice may have somehow compensated for the increase in cholesterol. Apoe^shl^ mice have high plasma cholesterol levels and are therefore termed spontaneous hyperlipidaemia (SHL) mice [13]. However, the age-related changes in blood cholesterol levels in Apoe^shl^ mice have not been clarified. The results of this study showed that also aged Apoe^shl^ mice have increased serum cholesterol levels. Plasma lipid levels are consistently elevated in Apoe^shl^ mice [13]. This can independently cause synaptic dysfunction and cognitive impairment [49]. The brain is the organ with the most cholesterol, containing approximately 25% of the total body cholesterol [50,51]. Impaired cholesterol homeostasis may promote neuroinflammatory responses and worsening cognitive dysfunction, and has been associated with various psychiatric disorders, including mood disorders [52,53]. Dysregulation of brain cholesterol homeostasis is associated with several chronic neurological and neurodegenerative diseases [54]. It has been reported that a type of cholesterol accumulates in the brain of AD model mice, leading to brain damage and behavioural changes [55,56]. Indeed, promoting the excretion of cholesterol from the brain has been shown to alleviate behavioural abnormalities. Further studies are needed to investigate brain cholesterol deposition in Apoe^shl^ mice. Another mechanism by which the loss of ApoE may lead to behavioural abnormalities is through its effects on the vasculature. Vascular dysfunction and atherosclerosis occur early in ApoE-deficient mice, leading to decreased cerebral blood flow and impaired autonomic control of the cerebral vasculature [26,57]. Further studies are needed to investigate the mechanisms underlying the abnormal behaviour in Apoe^shl^ mice.

ApoE-deficient female mice are more susceptible to cognitive dysfunction than male mice [58]. Moreover, the risk of AD is higher in women than in men [59,60]. In the present study, we performed experiments on male Apoe^shl^ mice. We need to perform the same behavioural experiments in female Apoe^shl^ mice. Compared to experiments with male mice, experiments with female mice may yield more significant differences. Unlike humans, mice express only one type of ApoE [61]. Therefore, there is still room for discussion regarding the usefulness of the results of this Apoe^shl^ mouse experiment as a human disease model.

## 5 Conclusions

The present study provides evidence that 7- and 11-month-old Apoe^shl^ mice exhibit different behavioural abnormalities. Moreover, Apoe^shl^ mice show behavioural abnormalities that varied depending on their age that differ from those observed in wild-type mice. This study demonstrates that Apoe^shl^ mice are useful not only as a model for investigating the function of ApoE in hyperlipidaemia but also as a model for investigating the function of ApoE in the CNS. To investigate the function of ApoE in the CNS, histological and biochemical analysis of Apoe^shl^ mice is required.

## References

[1] Pitas RE, Boyles JK, Lee SH, Hui D, Weisgraber KH. Lipoproteins and their receptors in the central nervous system. Characterization of the lipoproteins in cerebrospinal fluid and identification of apolipoprotein B,E(LDL) receptors in the brain. J Biol Chem. 1987;262:14352–60. 3115992

[2] Genin E, Hannequin D, Wallon D, Sleegers K, Hiltunen M, Combarros O, et al. APOE and Alzheimer disease: a major gene with semi-dominant inheritance. Mol Psychiatry. 2011;16:903–7. 10.1038/mp.2011.52 PMC3162068 21556001

[3] Caselli RJ, Dueck AC, Osborne D, Sabbagh MN, Connor DJ, Ahern GL, et al. Longitudinal modeling of age-related memory decline and the APOE epsilon4 effect. New Engl J Med. 2009;361:255–63. 10.1056/NEJMoa0809437 PMC2928998 19605830

[4] Shinohara M, Kanekiyo T, Yang L, Linthicum D, Shinohara M, Fu Y, et al. APOE2 eases cognitive decline during aging: clinical and preclinical evaluations. Ann Neurol. 2016;79:758–74. 10.1002/ana.24628 PMC5010530 26933942

[5] Liu C-C, Kanekiyo T, Xu H, Bu G. Apolipoprotein E and Alzheimer disease: risk, mechanisms, and therapy. Nat Rev Neurol. 2013;9:106–18. 10.1038/nrneurol.2012.263 PMC3726719 23296339

[6] Mahley RW, Rall SC. Apolipoprotein E: far more than a lipid transport protein. Annu Rev Genom Hum Genet. 2000;1:507–37. 10.1146/annurev.genom.1.1.507 11701639

[7] Boyles JK, Pitas RE, Wilson E, Mahley RW, Taylor JM. Apolipoprotein E associated with astrocytic glia of the central nervous system and with non myelinating glia of the peripheral nervous system. J Clin Investig. 1985;76:1501–13. 10.1172/JCI112130 PMC424114 3932467

[8] Boyles JK, Zoellner CD, Anderson LJ, Kosik LM, Pitas RE, Weisgraber KH, et al. A role for apolipoprotein E, apolipoprotein A-1, and low density lipoprotein receptors in cholesterol transport during regeneration and remyelination of rat sciatic nerve. J Clin Investig. 1989;83:1015–31. 10.1172/JCI113943 PMC303779 2493483

[9] Fuentes D, Fernández N, García Y, García T, Morales AR, Menéndez R. Age-related changes in the behavior of apolipoprotein E knockout mice. Behav Sci (Basel). 2018;8:33. 10.3390/bs8030033 PMC5867486 29510495

[10] Masliah E, Mallory M, Ge N, Alford M, Veinbergs I, Roses AD. Neurodegeneration in the central nervous system of apoE-deficient mice. Exp Neurol. 1995;136:107–22. 10.1006/exnr.1995.1088 7498401

[11] Fisher A, Brandeis R, Chapman S, Pittel Z, Michaelson DM. M1 muscarinic agonist treatment reverses cognitive and cholinergic impairments of apolipoprotein E-deficient mice. J Neurochem. 1998;70:1991–7. 10.1046/j.1471-4159.1998.70051991.x 9572284

[12] Anderson R, Higgins GA. Absence of central cholinergic deficits in ApoE knockout mice. Psychopharmacology (Berlin). 1997;132:135–44. 10.1007/s002130050329 9266610

[13] Matsushima Y, Hayashi S, Tachibana M. Spontaneously hyperlipidemic (SHL)mice: Japanese wild mice with apolipoprotein E deficiency. Mamm Genome. 1999;10:325–57. 10.1007/s003359901000 10087291

[14] Matsushima Y, Sakurai T, Ohoka A, Ohnuki T, Tada N, Asoh Y, et al. Four strains of spontaneously hyperlipidemic (SHL) mice: phenotypic distinctions determined by genetic backgrounds. J Atheroscler Thromb. 2001;8:71–9. 10.5551/jat1994.8.71 11866033

[15] Ueno H, Takahashi Y, Murakami S, Wani K, Miyazaki T, Matsumoto Y, et al. Comprehensive behavioral study of C57BL/6.KOR-ApoEshl mice. Transl Neurosci. 2023;14:20220284. 10.1515/tnsci-2022-0284 PMC10314129 37396111

[16] Mora F, Segovia G, del Arco A. Aging, plasticity and environmental enrichment: structural changes and neurotransmitter dynamics in several areas of the brain. Brain Res Rev. 2007;55:78–88. 10.1016/j.brainresrev.2007.03.011 17561265

[17] Rosenzweig ES, Barnes CA. Impact of aging on hippocampal function: plasticity, network dynamics, and cognition. Prog Neurobiol. 2003;69:143–79. 10.1016/s0301-0082(02)00126-0 12758108

[18] Perna G, Iannone G, Alciati A, Caldirola D. Are anxiety disorders associated with accelerated aging? A focus on neuroprogression. Neural Plast. 2016;2016:8457612. 10.1155/2016/8457612 PMC4736204 26881136

[19] Shoji H, Takao K, Hattori S, Miyakawa T. Age-related changes in behavior in C57BL/6J mice from young adulthood to middle age. Mol Brain. 2016;9:11. 10.1186/s13041-016-0191-9 PMC4730600 26822304

[20] Botton PH, Pochmann D, Rocha AS, Nunes F, Almeida AS, Marques DM, et al. Aged mice receiving caffeine since adulthood show distinct patterns of anxiety-related behavior. Physiol Behav. 2017;170:47–53. 10.1016/j.physbeh.2016.11.030 27890589

[21] Benice TS, Rizk A, Kohama S, Pfankuch T, Raber J. Sex-differences in age-related cognitive decline in C57BL/6J mice associated with increased brain microtubule-associated protein 2 and synaptophysin immunoreactivity. Neuroscience. 2006;137:413–23. 10.1016/j.neuroscience.2005.08.029 16330151

[22] Yanai S, Endo S. Functional aging in male C57BL/6J mice across the life-span: a systematic behavioral analysis of motor, emotional, and memory function to define an aging phenotype. Front Aging Neurosci. 2021;13:697621. 10.3389/fnagi.2021.697621 PMC8365336 34408644

[23] Matsumoto K, Yokoyama S, Gato N. Hypolipidemic effect of young persimmon fruit in C57BL/6.KOR-ApoEshl mice. Biosci Biotechnol Biochem. 2008;72:2651–9. 10.1271/bbb.80319 18838807

[24] Takeda S, Hirota R, Teradaira S, Takeda-Imoto M, Watanabe K, Toda A, et al. Cannabidiol-2’,6’-dimethyl ether stimulates body weight gain in apolipoprotein E-deficient BALB/c. KOR/Stm Slc-Apoe(shl) mice. J Toxicol Sci. 2015;40:739–43. 10.2131/jts.40.739 26558454

[25] Plump AS, Smith JD, Hayek T, Aalto-Setälä K, Walsh A, Verstuyft JG, et al. Severe hypercholesterolemia and atherosclerosis in apolipoprotein E-deficient mice created by homologous recombination in ES cells. Cell. 1992;71:343–53. 10.1016/0092-8674(92)90362-g 1423598

[26] Zhang SH, Reddick RL, Piedrahita JA, Maeda N. Spontaneous hypercholesterolemia and arterial lesions in mice lacking apolipoprotein E. Science. 1992;258:468–71. 10.1126/science.1411543 1411543

[27] Smith JP, Hicks PS, Ortiz LR, Martinez MJ, Mandler RN. Quantitative measurement of muscle strength in the mouse. J Neurosci Methods. 1995;62:15–9. 10.1016/0165-0270(95)00049-6 8750080

[28] Buchman AS, Boyle PA, Wilson RS, Beck TL, Kelly JF, Bennett DA. Apolipoprotein E e4 allele is associated with more rapid motor decline in older persons. Alzheimer Dis Assoc Disord. 2009;23:63–9. 10.1097/wad.0b013e31818877b5 PMC2662708 19266700

[29] Shiotsuki H, Yoshimi K, Shimo Y, Funayama M, Takamatsu Y, Ikeda K, et al. A rotarod test for evaluation of motor skill learning. J Neurosci Methods. 2010;189:180–5. 10.1016/j.jneumeth.2010.03.026 20359499

[30] van der Flier WM, Staekenborg S, Pijnenburg YA, Gillissen F, Romkes R, Kok A, et al. Apolipoprotein E genotype influences presence and severity of delusions and aggressive behaviour in Alzheimer disease. Dement Geriatr Cogn Disord. 2007;3:42–6. 10.1159/000096682 17077632

[31] Litherland MT. Behavioral aspects of apolipoprotein E knockout mice. Masters theses. 2003. p. 1424.

[32] McGeer PL, McGeer EG. Inflammation and the degenerative diseases of aging. Ann N Y Acad Sci. 2004;1035:104–16. 10.1196/annals.1332.007 15681803

[33] Gorina YV, Komleva YK, Lopatina OL, Volkova VV, Chernykh AI, Shabalova AA, et al. The battery of tests for experimental behavioral phenotyping of aging animals. Adv Gerontol. 2017;7:137–42. 28557390

[34] Martin-Aragon S, Villar A, Benedi J. Age-dependent effects of esculetin on mood-related behavior and cognition from stressed mice are associated with restoring brain antioxidant status. Prog Neuropsychopharmacol Biol Psychiatry. 2016;65:1–16. 10.1016/j.pnpbp.2015.08.007 26290950

[35] Zhu D, Montagne A, Zhao Z. Alzheimer’s pathogenic mechanisms and underlying sex difference. Cell Mol Life Sci. 2021;78:4907–20. 10.1007/s00018-021-03830-w PMC8720296 33844047

[36] Raber J, Akana SF, Bhatnagar S, Dallman MF, Wong D, Mucke L. Hypothalamic-pituitary-adrenal dysfunction in Apoe−/−mice: possible role in behavioral and metabolic alterations. J Neurosci. 2000;20:2064–71. 10.1523/JNEUROSCI.20-05-02064.2000 PMC6772921 10684907

[37] de Magalhães JP, Wuttke D, Wood SH, Plank M, Vora C. Genome–environment interactions that modulate aging: powerful targets for drug discovery. Pharmacol Rev. 2012;64:88–101. 10.1124/pr.110.004499 PMC3250080 22090473

[38] Rando TA, Wyss-Coray T. Asynchronous, contagious and digital aging. Nat Aging. 2021;1:29–35. 10.1038/s43587-020-00015-1 PMC8248898 34223194

[39] Raber J. Role of apolipoprotein E in anxiety. Neural Plast. 2007;2007:091236. 10.1155/2007/91236 PMC1940061 17710250

[40] Seibenhener ML, Wooten MC. Use of the open field maze to measure locomotor and anxiety-like behavior in mice. J Vis Exp. 2015;6:e52434. 10.3791/52434 PMC4354627 25742564

[41] Fahlström A, Yu Q, Ulfhake B. Behavioral changes in aging female C57BL/6 mice. Neurobiol Aging. 2011;32:1868–80. 10.1016/j.neurobiolaging.2009.11.003 20005598

[42] Mauch DH, Nagler K, Schumacher S, Goritz C, Muller EC, Otto A, et al. CNS synaptogenesis promoted by glia-derived cholesterol. Science. 2001;294:1354–7. 10.1126/science.294.5545.1354 11701931

[43] Pfrieger FW. Cholesterol homeostasis and function in neurons of the central nervous system. Cell Mol Life Sci. 2003;60:1158–71. 10.1007/s00018-003-3018-7 PMC11138592 12861382

[44] Anderson R, Barnes JC, Bliss TVP, Cain DP, Cambon K, Davies HA, et al. Behavioural, physiological and morphological analysis of a line of apolipoprotein E knockout mouse. Neuroscience. 1998;85:93–110. 10.1016/s0306-4522(97)00598-8 9607706

[45] Bour A, Grootendorst J, Vogel E, Kelche C, Dodart J-C, Bales K, et al. Middle-aged human apoE4 targeted-replacement mice show retention deficits on a wide range of spatial memory tasks. Behav Brain Res. 2008;93:174–82. 10.1016/j.bbr.2008.05.008 18572260

[46] Raber J, Wong D, Buttini M, Orth M, Bellosta S, Pitas RE, et al. Isoform specific effects of human apolipoprotein E on brain function revealed in ApoE knockout mice: Increased susceptibility of females. Proc Natl Acad Sci USA. 1998;95:10914–19. 10.1073/pnas.95.18.10914 PMC27995 9724804

[47] Grootendorst J, de Kloet ER, Vossen C, Dalm S, Oitzl MS. Repeated exposure to rats has persistent genotype-dependent effects on learning and locomotor activity of apolipoprotein E knockout and C57Bl/6 mice. Behav Brain Res. 2001;125:249–59. 10.1016/s0166-4328(01)00294-7 11682116

[48] Feng F, Lu SS, Hu CY, Gong FF, Qian ZZ, Yang HY, et al. Association between apolipoprotein E gene polymorphisms and depression. J Clin Neurosci. 2015;22:1232–8. 10.1016/j.jocn.2015.02.012 25979253

[49] Anstey KJ, Lipnicki DM, Low LF. Cholesterol as a risk factor for dementia and cognitive decline: a systematic review of prospective studies with meta-analysis. Am J Geriatr Psychiatry. 2008;16:343–54. 10.1097/JGP.0b013e31816b72d4 18448847

[50] Dietschy JM, Turley SD. Cholesterol metabolism in the central nervous system during early development and in the mature animal. J Lipid Res. 2004;45:1375–97. 10.1194/jlr.R400004-JLR200 15254070

[51] Dietschy JM. Central nervous system: cholesterol turnover, brain development and neurodegeneration. Biol Chem. 2009;390:287–93. 10.1515/BC.2009.035 PMC3066069 19166320

[52] Chen Y, Jiang T, Chen P, Ouyang J, Xu G, Zeng Z, et al. Emerging tendency towards autoimmune process in major depressive patients: a novel insight from Th17 cells. Psychiatry Res. 2011;188:224–30. 10.1016/j.psychres.2010.10.029 21129782

[53] Lassale C, Batty GD, Baghdadli A, Jacka F, Sánchez-Villegas A, Kivimäki M, et al. Healthy dietary indices and risk of depressive outcomes: a systematic review and meta-analysis of observational studies. Mol Psychiatry. 2019;24:965–86. 10.1038/s41380-018-0237-8 PMC6755986 30254236

[54] Valenza M, Cattaneo E. Emerging roles for cholesterol in Huntington’s disease. Trends Neurosci. 2011;34:474–86. 10.1016/j.tins.2011.06.005 21774998

[55] Martin MG, Ahmed T, Korovaichuk A, Venero C, Menchón SA, Salas I, et al. Constitutive hippocampal cholesterol loss underlies poor cognition in old rodents. EMBO Mol Med. 2014;6:902–17. 10.15252/emmm.201303711 PMC4119354 24878762

[56] Litvinchuk A, Suh JH, Guo JL, Lin K, Davis SS, Bien-Ly N, et al. Amelioration of Tau and ApoE4-linked glial lipid accumulation and neurodegeneration with an LXR agonist. Neuron. 2024;112:384–403. 10.1016/j.neuron.2023.10.023 PMC10922706 37995685

[57] Ayata C, Shin HK, Dileko ZE, Atochin DN, Kashiwagi S, Eikermann-Haerter K, et al. Hyperlipidemia disrupts cerebrovascular reflexes and worsens ischemic perfusion defect. J Cereb Blood Flow Metab. 2013;33:954–62. 10.1038/jcbfm.2013.38 PMC3677117 23486293

[58] Raber J, Wong D, Yu GQ, Buttini M, Mahley RW, Pitas RE, et al. Apolipoprotein E and cognitive performance. Nature. 2000;404:352–4. 10.1038/35006165 10746713

[59] Ruitenberg A, Ott A, van Swieten JC, Hofman A, Breteler MM. Incidence of dementia: does gender make a difference? Neurobiol Aging. 2001;22:575–80. 10.1016/s0197-4580(01)00231-7 11445258

[60] Hebert LE, Scherr PA, Bienias JL, Bennett DA, Evans DA. Alzheimer disease in the US population: prevalence estimates using the 2000 census. Arch Neurol. 2003;60:1119–22. 10.1001/archneur.60.8.1119 12925369

[61] Kim J, Basak JM, Holtzman DM. The role of apolipoprotein E in Alzheimer’s disease. Neuron. 2009;63:287–303. 10.1016/j.neuron.2009.06.026 PMC3044446 19679070

